# Dose-Dependent Genome-Wide DNA Methylation Remodeling by Metformin Modulates Doxorubicin Sensitivity in Cardiac Cells

**DOI:** 10.3390/epigenomes10030044

**Published:** 2026-07-03

**Authors:** Mahmoud Abu Shayeb, Nagham N. Hendi, Georges Nemer, Hana Hammad, Malek Zihlif, Heba Saadeh, Heba Mansour

**Affiliations:** 1 Department of Pathology, Microbiology and Forensic Medicine, Faculty of Medicine, The University of Jordan, Amman 11942, Jordan; mhm8220939@ju.edu.jo; 2 Department of Clinical Pharmacy & Therapeutics, Faculty of Pharmacy, Applied Science Private University (ASU), Amman 11937, Jordan; n_hendi@asu.edu.jo; 3 College of Health and Life Sciences, Hamad Bin Khalifa University, Qatar Foundation, Doha P.O. Box 34110, Qatar; gnemer@hbku.edu.qa; 4 Department of Biological Sciences, Faculty of Science, The University of Jordan, Amman 11942, Jordan; hhammad@ju.edu.jo (H.H.); hebamansour1989@gmail.com (H.M.); 5 Department of Pharmacology, Faculty of Medicine, The University of Jordan, Amman 11942, Jordan; 6 Department of Computer Science, King Abdullah II School of Information Technology, The University of Jordan, Amman 11942, Jordan; heba.saadeh@ju.edu.jo

**Keywords:** metformin, doxorubicin, cardiotoxicity, DNA methylation, epigenetics, bisulfite sequencing

## Abstract

**Background/Objectives:** Doxorubicin (DOX) is an effective chemotherapeutic agent, but its clinical use is limited by dose-dependent cardiotoxicity. Emerging evidence suggests that epigenetic dysregulation, particularly altered DNA methylation, contributes to DOX-induced cardiac injury. Metformin has been reported to exert cardiometabolic and epigenetic regulatory effects. This study investigated genome-wide DNA methylation changes induced by chronic metformin exposure and their effects on doxorubicin sensitivity in H9c2 cardiomyoblast cells. **Methods:** Genome-wide DNA methylation changes induced by chronic metformin exposure were investigated in H9c2 cardiomyoblast cells using whole-genome bisulfite sequencing (WGBS). Cells were treated with metformin (0.7–2.8 mM) for four months prior to DOX exposure. Cellular sensitivity to DOX was evaluated using MTT-based dose–response analysis and IC_50_ estimation. **Results:** DOX reduced cell viability (IC_50_ = 0.164 µM). Chronic metformin pre-treatment produced a dose-dependent rightward shift in DOX dose–response curves, increasing IC_50_ values to 0.21, 0.289, and 0.51 µM at 0.7, 1.4, and 2.8 mM metformin, respectively. WGBS revealed distinct separation between treatment groups in principal component analysis. Significant methylation changes (adjusted *p*-value < 0.05) were identified in genes related to oxidative stress, mitochondrial function, apoptosis, and chromatin regulation. **Conclusions:** Chronic metformin exposure induces dose-dependent genome-wide DNA methylation remodeling in cardiac cells and is associated with altered cellular sensitivity to doxorubicin. These findings suggest that metabolic modulation by metformin may influence epigenetic regulation and cellular stress responses relevant to chemotherapy-induced cardiotoxicity.

## 1. Introduction

Doxorubicin (DOX) is a widely used anthracycline chemotherapeutic agent effective against various malignancies, including leukemia, lymphoma, and breast cancer. However, its clinical utility is limited by cumulative, dose-dependent cardiotoxicity that may progress to cardiomyopathy and heart failure in long-term cancer survivors [1,2]. Mechanistically, DOX-induced cardiotoxicity involves oxidative stress, mitochondrial dysfunction, lipid peroxidation, and DNA damage, with cardiomyocytes being particularly vulnerable due to their high mitochondrial content and limited antioxidant capacity [3].

Doxorubicin-induced cardiotoxicity is mediated by multiple interconnected mechanisms, including oxidative stress, mitochondrial dysfunction, inflammation, and ferroptosis. Doxorubicin promotes excessive production of reactive oxygen species (ROS), leading to lipid peroxidation, DNA damage, and activation of cell death pathways. Mitochondrial dysfunction further exacerbates energy imbalance and apoptotic signaling, while inflammatory responses contribute to long-term cardiac remodeling. Ferroptosis, an iron-dependent form of regulated cell death associated with lipid peroxidation, has also been implicated in doxorubicin-induced cardiac injury.

Beyond these classical mechanisms, increasing evidence suggests that epigenetic dysregulation contributes to DOX-induced cardiac injury. DNA methylation, mediated by DNA methyltransferases (*DNMT1*, *DNMT3A*, and *DNMT3B*), regulates chromatin structure and gene expression and may establish long-term epigenetic memory in response to toxic stress [4,5]. Aberrant methylation patterns have been implicated in cardiomyopathy and mitochondrial dysfunction, suggesting that chemotherapy-induced epigenetic alterations may contribute to delayed cardiac remodeling.

Metformin, a first-line antidiabetic agent, has gained attention for its pleiotropic cardiometabolic effects [6]. Metformin was selected due to its well-established cardiometabolic and cytoprotective effects. Through activation of AMP-activated protein kinase (AMPK) and modulation of mitochondrial function, metformin reduces oxidative stress and improves cellular energy homeostasis [7]. Emerging evidence also indicates that metformin can modulate epigenetic regulation by influencing DNMT expression and altering DNA methylation patterns in various cell types [8].

Although metformin has been investigated for its cardioprotective properties, its impact on genome-wide DNA methylation remodeling in cardiac cells remains incompletely characterized. Understanding whether chronic metformin exposure induces stable epigenetic reprogramming may provide mechanistic insight into how metabolic modulation influences cellular responses to chemotherapeutic stress.

Therefore, the present study aimed to characterize genome-wide DNA methylation changes induced by chronic metformin exposure in H9c2 cardiomyoblast cells using whole-genome bisulfite sequencing (WGBS). In parallel, we evaluated whether long-term metformin pre-treatment alters cellular sensitivity to doxorubicin, as assessed by dose–response and IC_50_ analysis.

Recent studies have further highlighted the role of epigenetic modulation in doxorubicin-induced cardiotoxicity, including alterations in DNA methylation, histone modifications, and non-coding RNA regulation [9,10,11,12]. In parallel, metformin has been increasingly recognized for its epigenetic effects, including modulation of DNA methyltransferase activity and genome-wide methylation patterns [13]. These findings support the concept that metabolic interventions may influence epigenetic responses to chemotherapeutic stress.

## 2. Results

### 2.1. Selection of Experimental Conditions for Genome-Wide DNA Methylation Analysis

**Figure 1:**
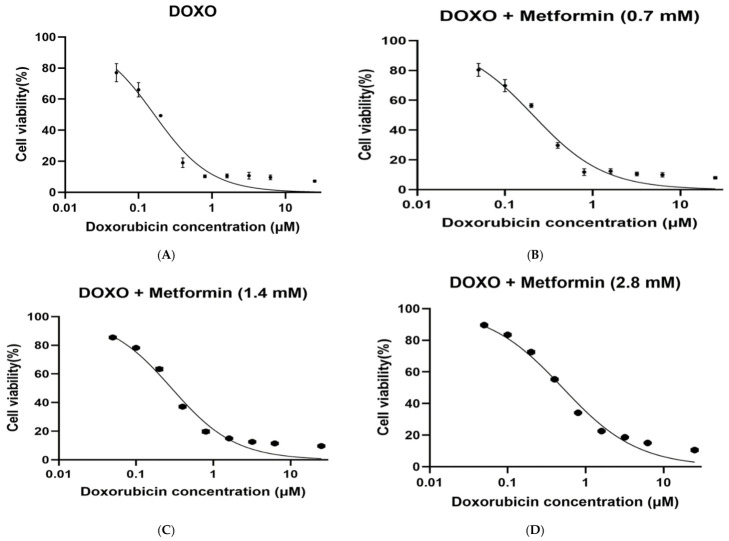
Metformin (MET) pre-treatment shifts doxorubicin (DOX) dose–response curves in H9c2 cardiomyoblast cells. (**A**) DOX alone, (**B**) DOX and 0.7 mM MET, (**C**) DOX and 1.4 mM MET, and (**D**) DOX and 2.8 mM MET.

Doxorubicin (DOX) induced a dose-dependent reduction in H9c2 cardiomyoblast cell viability, as demonstrated by nonlinear regression analysis using a four-parameter logistic (4PL) model (Figure 1A–D). The calculated IC_50_ value for DOX alone was 0.164 µM. Pre-treatment with metformin produced a concentration-dependent rightward shift in DOX dose–response curves. The IC_50_ values increased to approximately 0.21 µM, 0.289 µM, and 0.51 µM following chronic exposure to 0.7 mM, 1.4 mM, and 2.8 mM metformin, respectively. Comparison of fitted log IC_50_ values using the extra sum-of-squares F test demonstrated that metformin produced a statistically significant rightward shift in the doxorubicin dose–response curve at 1.4 mM (*p* < 0.05) and 2.8 mM (*p* < 0.01), whereas the 0.7 mM group did not show a statistically significant shift (*p* > 0.05). These findings indicate reduced DOX cytotoxicity after long-term metformin exposure in a dose-dependent manner.

The absence of a statistically significant shift at 0.7 mM suggests a threshold-dependent response, where lower metformin concentrations may not be sufficient to induce measurable changes in cellular sensitivity to doxorubicin under the experimental conditions used.

Cells were exposed to increasing concentrations of doxorubicin (0.05, 0.1, 0.2, 0.4, 0.8, 1.6, 3.2, 6.25, and 25 µM), and viability was assessed by MTT assay and normalized to untreated control (100%). Curves were fitted using nonlinear regression (4PL model; top and bottom constrained to 100% and 0%, respectively). IC_50_ values were 0.164 µM for DOX alone, and approximately 0.21, 0.289, and 0.51 µM for 0.7, 1.4, and 2.8 mM MET pre-treatment, respectively. Data represent mean ± SD from four independent biological replicates (n = 4), each performed in triplicate technical wells. Cells were seeded at 5 × 10^3^ cells/well.

### 2.2. Global Patterns of DNA Methylation in Treatment Groups

Genome-wide DNA methylation analysis using 1 kb genomic tiles was performed to evaluate global epigenetic patterns across experimental groups. PCA revealed clear separation between control and metformin-treated H9c2 cells. Control samples clustered tightly, indicating high intra-group consistency of baseline methylation profiles.

The first principal component (PC1) accounted for the largest proportion of variance and separated metformin-treated samples from controls. High-dose metformin samples formed a distinct cluster, while low-dose samples occupied an intermediate position between control and high-dose groups, demonstrating a dose-dependent shift in global DNA methylation patterns.

These findings indicate that chronic metformin exposure induces dose-dependent, genome-wide alterations in DNA methylation in H9c2 cardiomyoblast cells.

**Figure 2:**
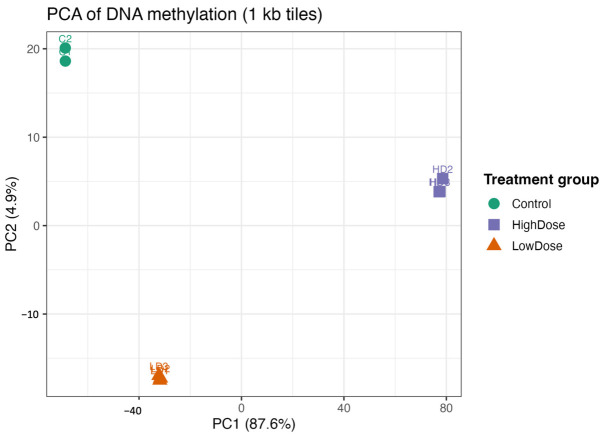
Principal component analysis reveals dose-dependent genome-wide DNA methylation changes following chronic metformin exposure. Green circles represent control samples, purple squares represent low-dose MET-treated cells, and orange triangles represent high-dose MET-treated H9c2 cells.

PCA of genome-wide DNA methylation levels calculated from 1 kb genomic tiles in H9c2 cardiomyoblast cells. Samples from control, low-dose-MET-treated, and high-dose-MET-treated groups show clear separation along the first two principal components (Figure 2), indicating distinct global methylation patterns. The clustering demonstrates dose-dependent epigenetic reprogramming induced by metformin at the genome-wide level.

### 2.3. Identification of Differentially Methylated Regions in Metformin-Treated Cells

Differential methylation analysis was performed to identify genomic regions significantly altered following metformin treatment. Volcano plot visualization demonstrated both hypermethylated and hypomethylated regions compared with control cells. A substantial number of regions exceeded predefined statistical significance thresholds (FDR < 0.05), indicating that metformin induces widespread and bidirectional DNA methylation changes rather than a uniform global shift. These results support a significant epigenetic effect of metformin in cardiac cells.

### 2.4. Quality Assessment of Whole-Genome Bisulfite Sequencing Data

Prior to differential methylation analysis, sequencing quality and methylation data integrity were evaluated across all samples. Genome-wide β-value distributions exhibited the expected bimodal pattern, with enrichment near unmethylated (β ≈ 0) and fully methylated (β ≈ 1) CpG sites, confirming efficient bisulfite conversion and reliable methylation calling. Sequencing coverage analysis demonstrated adequate and uniform read depth across CpG sites, supporting the robustness of downstream differential methylation analysis. Detailed genome-wide β-value distributions and CpG coverage plots are provided in Supplementary Figure S3.

To assess potential batch effects, principal component analysis (PCA) and clustering patterns were evaluated across all samples. No evidence of batch-related clustering was observed, indicating that technical variation did not significantly influence the methylation profiles.

### 2.5. High-Dose Metformin Induces Differential DNA Methylation

**Figure 3:**
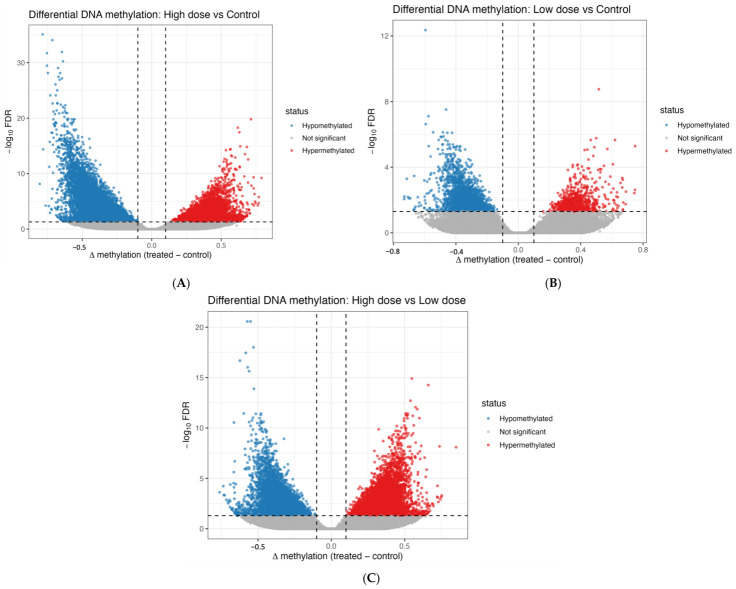
Differential DNA methylation analysis identifies hyper- and hypomethylated regions across metformin treatment conditions. (**A**) Volcano plot of differentially methylated regions (DMRs) in high-dose MET versus control; (**B**) low-dose MET versus control; and (**C**) high-dose MET versus low-dose MET. The *x*-axis represents Δβ (difference in methylation levels between groups), and the *y*-axis represents −log10 FDR-adjusted *p*-values. Each point represents a DMR, with significant regions defined by FDR < 0.05 and the applied Δβ threshold.

Differential methylation analysis comparing high-dose metformin-treated cells with untreated controls revealed a substantial number of significantly altered genomic regions (Figure 3A). Both hypermethylated and hypomethylated regions were identified, confirming bidirectional epigenetic remodeling rather than a global directional shift.

Hypermethylated regions were enriched at positive Δβ values, whereas hypomethylated regions were enriched at negative Δβ values exceeding statistical significance thresholds (FDR < 0.05). These findings demonstrate a pronounced genome-wide epigenetic impact of high-dose metformin in H9c2 cardiomyoblast cells.

### 2.6. Differential DNA Methylation Following Low-Dose Metformin Exposure

Comparison of low-dose metformin-treated cells with untreated controls identified both hypermethylated and hypomethylated regions, indicating bidirectional epigenetic modulation (Figure 3B). However, the number of significantly altered regions and the magnitude of methylation changes were markedly lower than those observed in the high-dose group (Figure 3C). These results demonstrate a dose-dependent epigenetic response, with low-dose metformin inducing comparatively modest genome-wide DNA methylation alterations.

### 2.7. Hierarchical Clustering Analysis of Differentially Methylated Regions

**Figure 4:**
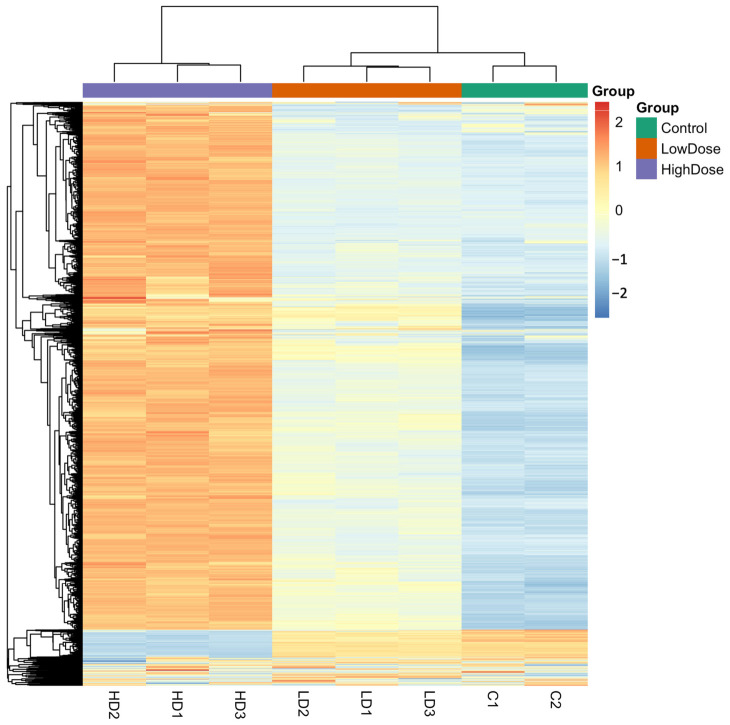
Hierarchical clustering of differentially methylated regions demonstrates dose-dependent epigenetic divergence between treatment groups. Hierarchical clustering heatmap of differentially methylated 1 kb-genomic tiles in H9c2 cardiomyoblast cells treated with low-dose and high-dose metformin compared with untreated controls. Rows represent genomic regions, and columns represent individual samples. Methylation levels are displayed as scaled β-values (z-score), with red indicating higher methylation and blue indicating lower methylation. Unsupervised clustering reveals a clear separation of samples according to treatment group, demonstrating a dose-dependent epigenetic reprogramming effect of metformin on the cardiac DNA methylation landscape.

Unsupervised hierarchical clustering of differentially methylated genomic regions demonstrated clear grouping of samples according to treatment condition (Figure 4). Control samples clustered tightly, reflecting stable baseline methylation patterns. High-dose metformin-treated samples formed a distinct cluster characterized by pronounced methylation alterations, whereas low-dose samples exhibited an intermediate clustering pattern. This clustering behavior confirms a dose-dependent epigenetic effect of metformin on global DNA methylation in H9c2 cardiomyoblast cells.

### 2.8. Dose-Dependent Epigenetic Responses to Metformin

To confirm that metformin alone did not exert cytotoxic effects under the selected experimental conditions, H9c2 cells were treated with metformin (0.7, 1.4, and 2.8 mM) in the absence of doxorubicin. No statistically significant reduction in cell viability was observed compared with vehicle-treated controls (*p* > 0.05), as shown in Supplementary Figure S2

Furthermore, during both acute (24 h) exposure and the four-month chronic treatment period, no evidence of sustained growth arrest, morphological abnormalities, or viability reduction was observed in metformin-treated cultures compared with passage-matched controls. These findings confirm that the selected metformin concentrations were non-cytotoxic and appropriate for evaluating epigenetic and modulatory effects.

### 2.9. Comparison of the Most and Least Epigenetically Affected Genes in LD and HD Groups

Comparison of genes showing the highest and lowest numbers of differentially methylated regions (DMRs) in low-dose metformin (LD)-treated H9c2 cells, including methylation direction and associated biological functions, are summarized in Table 1 and Table 2, respectively. Additional details are provided in the Supplementary Materials. Although some of the identified genes (e.g., *GAB2*, *TENM4*) are not classically associated with cardiomyocyte-specific functions, they are involved in key cellular signaling pathways such as PI3K/AKT, cell survival, and stress response mechanisms. These pathways may indirectly influence cardiomyocyte biology and cellular responses to toxic stress.

### 2.10. Pathway Changes After Metformin Treatment for Low and High Doses

**Figure 5:**
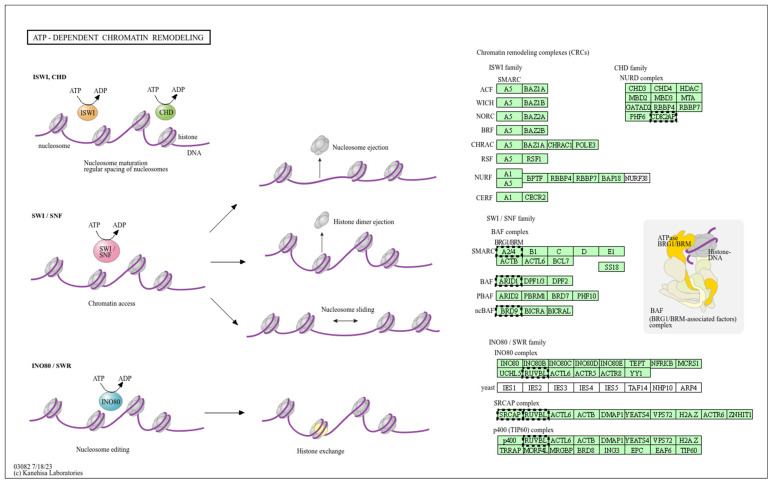
Enrichment of ATP-dependent chromatin remodeling complexes associated with low-dose metformin treatment. The schematic illustrates the major families of ATP-dependent chromatin remodeling complexes (CRCs), including ISWI, CHD (NuRD), SWI/SNF (BAF/PBAF), and INO80/SWR, which regulate chromatin structure through nucleosome sliding, spacing, ejection, histone dimer removal, and histone variant exchange. Pathway enrichment analysis was performed using DAVID [14].

Pathway enrichment analysis in the low-dose metformin group identified significant modulation of calcium signalling and related stress–response pathways (Figure 5). A detailed schematic representation of the calcium signalling pathway (low dose) is provided in Supplementary Figure S4. In addition, phosphatidylinositol (PI) signalling pathways were enriched in the low-dose group, and a detailed pathway map is provided in Supplementary Figure S5. Enrichment analysis also indicated involvement of thermogenesis and mitochondrial energy metabolism pathways in the low-dose group, illustrated in Supplementary Figure S6. These processes influence chromatin accessibility and may interact with DNA methylation machinery in response to metabolic modulation.

**Figure 6:**
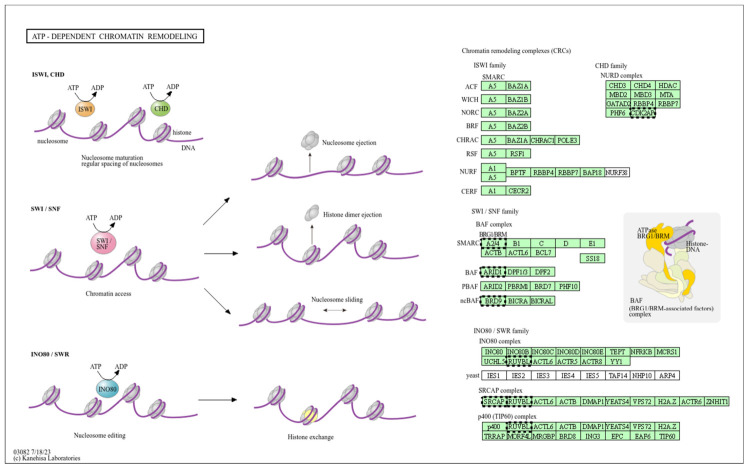
Chromatin remodeling pathways enriched following high-dose metformin-induced epigenetic remodeling. The schematic highlights key ATP-dependent chromatin remodelling families and their regulatory roles in chromatin accessibility and transcriptional control. Pathway enrichment analysis was performed using DAVID [14].

In the high-dose metformin group, pathway enrichment analysis revealed more pronounced modulation of calcium signalling pathways compared with low-dose treatment (Figure 6). A detailed schematic representation of the calcium signalling pathway (high dose) is provided in Supplementary Figure S7. In addition, phosphatidylinositol (PI) signalling pathways were significantly enriched in the high-dose group, and a detailed pathway map is provided in Supplementary Figure S8. Enrichment analysis also demonstrated involvement of thermogenesis and mitochondrial energy metabolism pathways in the high-dose group, illustrated in Supplementary Figure S9. High-dose metformin treatment was associated with enrichment of genes related to chromatin remodeling pathways, suggesting dose-dependent epigenetic modulation mechanisms.

## 3. Discussion

The present study provides genome-wide evidence that chronic metformin exposure induces dose-dependent DNA methylation remodeling that alters doxorubicin sensitivity in cardiomyoblast cells. Using WGBS, we identified that low- and high-dose treatments produced bidirectional DNA methylation changes; however, the magnitude and number of differentially methylated regions were substantially greater in the high-dose group.

PCA revealed a clear separation between control, low-dose, and high-dose metformin-treated groups, supporting the existence of stable epigenetic reprogramming following prolonged metabolic modulation. The progressive clustering pattern observed across treatment groups further suggests a dose-dependent epigenetic gradient. These findings are consistent with previous reports demonstrating that metformin modulates DNA methyltransferase activity and influences global methylation landscapes through AMPK-dependent and AMPK-independent mechanisms [15].

Metformin exerts its biological effects primarily through activation of AMP-activated protein kinase (AMPK), a key regulator of cellular energy homeostasis. Metformin inhibits mitochondrial complex I, leading to an increase in intracellular AMP/ATP and ADP/ATP ratios, which in turn activates AMPK.

Activated AMPK regulates multiple downstream pathways involved in cellular metabolism and stress adaptation, including increased expression of small heterodimeric partner (SHP). AMPK activation improves mitochondrial function, reduces mitochondrial reactive oxygen species (ROS) production, and enhances cellular resistance to oxidative stress. In addition, AMPK inhibits the mechanistic target of rapamycin (mTOR) pathway, thereby reducing protein synthesis, promoting autophagy, and limiting cellular stress. These mechanisms collectively contribute to the cardioprotective effects of metformin observed in models of cardiac injury.

Previous studies have reported dose-dependent effects of metformin, in which lower concentrations exert protective effects, whereas higher concentrations may lead to diminished or altered responses. It has been suggested that excessive activation of AMPK at higher doses may suppress platelet-derived growth factor receptor (PDGFR) signaling, which plays a critical role in cell survival and stress adaptation. Reduced PDGFR signaling may impair the ability of cells to regulate reactive oxygen species and calcium homeostasis, thereby attenuating the protective effects of metformin. These findings highlight the complexity of metformin’s dose-dependent actions and suggest that optimal cardioprotective effects occur within a specific concentration range.

Differential methylation and integrative pathway analyses identified significant enrichment of genes involved in chromatin remodeling, calcium signaling, phosphatidylinositol signaling, and mitochondrial energy metabolism. These pathways are tightly interconnected with cardiomyocyte survival, stress adaptation, and metabolic homeostasis. Importantly, the DNA methylation changes observed in this study were not randomly distributed across the genome but were enriched in biologically relevant pathways associated with cardiomyocyte function and stress response. These include mitochondrial energy metabolism, oxidative stress regulation, calcium signaling, and chromatin remodeling pathways, all of which are known to play central roles in doxorubicin-induced cardiotoxicity.

The integration of genome-wide methylation profiling with functional dose–response analysis suggests that chronic metformin exposure may induce stable epigenetic reprogramming that alters cellular susceptibility to chemotherapeutic stress. While the present study does not establish direct causality, the observed association between epigenetic remodeling and altered doxorubicin sensitivity provides a mechanistically relevant framework for future investigations. Doxorubicin-induced cardiotoxicity has been associated with mitochondrial dysfunction, oxidative stress, calcium dysregulation, and epigenetic instability [2,3,9]. Therefore, the observed metformin-induced methylation changes in these metabolic pathways may reflect adaptive epigenetic reprogramming that modulates cellular stress responses.

Notably, high-dose metformin induced a greater number and magnitude of differentially methylated regions compared with low-dose exposure, further supporting a concentration-dependent epigenetic effect. This observation aligns with studies indicating that prolonged AMPK activation and metabolic reprogramming can lead to stable chromatin modifications and altered transcriptional landscapes [10,11]. Although direct transcriptomic integration was not performed in the present study, the pathway-level enrichment supports a biologically coherent pattern linking metabolic regulation with epigenetic remodeling.

Functionally, chronic metformin exposure was associated with a rightward shift in doxorubicin dose–response curves, reflected by increased IC_50_ values. While the current study did not directly profile doxorubicin-induced methylation changes, the association between altered methylation landscapes and modified cellular sensitivity suggests a potential epigenetic contribution to the observed phenotype [12,13,16]. It is important to emphasize that this does not establish causal reversal of doxorubicin-induced methylation but rather supports a modulatory association under the experimental conditions used.

An important consideration is whether metformin may attenuate the antitumor efficacy of doxorubicin. While the present study focuses on cardiomyocyte responses, previous studies have suggested that metformin may enhance or synergize with chemotherapeutic agents in certain cancer models through metabolic and epigenetic mechanisms. However, the potential impact of metformin on doxorubicin’s antitumor activity was not evaluated in this study and remains an important area for future investigation.

The enrichment of chromatin remodeling complexes among differentially methylated regions is particularly noteworthy. ATP-dependent chromatin remodelers interact closely with DNA methylation machinery to regulate chromatin accessibility and transcriptional stability [17]. Doxorubicin has been reported to disrupt chromatin organization and induce oxidative DNA damage, processes that may influence methylation patterns. Metformin-mediated stabilization of cellular energy homeostasis and reduction of oxidative stress may indirectly preserve chromatin integrity [15], thereby influencing epigenetic maintenance mechanisms. Importantly, these findings demonstrate an association rather than a direct causal relationship between metformin-induced methylation changes and altered doxorubicin sensitivity.

It should be noted that the metformin concentrations used in this study exceed clinically achievable plasma levels in humans. However, higher concentrations are commonly required in in vitro systems due to differences in drug uptake, metabolic conditions, and prolonged exposure duration. These experimental conditions are widely used to investigate cellular and epigenetic effects under controlled settings. Nevertheless, this represents a limitation when translating these findings to clinical contexts.

Metformin is generally considered safe in patients with stable heart failure and has been associated with improved clinical outcomes in some studies. However, it is contraindicated in patients with severe renal impairment due to the risk of lactic acidosis. Since doxorubicin-induced cardiotoxicity may lead to heart failure, careful clinical evaluation is required when considering metformin use in this context.

To our knowledge, this is among the first studies to combine chronic metformin exposure with whole-genome bisulfite sequencing to investigate epigenetic remodeling in cardiac cells in the context of doxorubicin sensitivity.

## 4. Limitations and Future Directions

Several limitations should be considered. First, WGBS was performed only in metformin-treated and control groups; therefore, genome-wide methylation changes induced directly by doxorubicin and their potential modulation by metformin were not evaluated. Second, functional validation experiments assessing oxidative stress, apoptosis, and mitochondrial activity were not included.

H9c2 cardiomyoblast cells are widely used as an in vitro model for studying cardiotoxicity due to their reproducibility, ease of culture, and responsiveness to doxorubicin-induced stress. However, it is important to note that H9c2 cells are derived from embryonic rat cardiac tissue and do not fully replicate the phenotype and functional properties of adult human cardiomyocytes, including contractility and metabolic complexity.

Future studies should incorporate genome-wide methylation profiling of DOX-only and DOX+MET treatment groups, combined with functional assays and transcriptomic integration to better define mechanistic links between epigenetic remodeling and cellular stress responses. Validation in human cardiomyocyte models and in vivo systems will also be important to strengthen translational relevance in cardio-oncology.

In addition, transcriptomic and proteomic validation of methylation-associated gene expression changes was not performed.

This represents a key limitation of the present study and should be addressed in future investigations.

## 5. Materials and Methods

### 5.1. Experimental Groups

H9c2 cells were allocated to the following experimental groups:

- Control (untreated): Baseline evaluation of cell health and the state of DNA methylation.
- Metformin alone: Cells were incubated with predetermined non-cytotoxic concentrations of metformin.
- DOX alone: Cells were incubated with DOX at its half-maximal inhibitory concentration (IC_50_) for 24 h.
- Metformin + DOX (pre-treatment): Cells had been pre-treated with metformin over the course of 4 months and then subjected to DOX.

Each experimental condition was performed in four independent biological replicates (*n* = 4). Within each biological replicate, treatments were assessed in triplicate technical wells.

Each replicate represents an independent biological experiment performed on separately cultured cells.

### 5.2. Cell Culture

The H9c2 rat cardiomyoblast cell line (ATCC^®^ CRL-1446™, RRID: CVCL_0286) was obtained from the American Type Culture Collection (ATCC, Manassas, VA, USA). Cells were cultured in Dulbecco’s Modified Eagle Medium (DMEM; Gibco, Thermo Fisher Scientific, Waltham, MA, USA; Cat. No. 11965-092) supplemented with 10% fetal bovine serum (FBS; Gibco; Cat. No. 16000-044), 1% penicillin–streptomycin (100 U/mL penicillin and 100 µg/mL streptomycin; Gibco; Cat. No. 15140-122), and 1% L-glutamine (Gibco; Cat. No. 25030-081). Cells were maintained at 37 °C in a humidified incubator containing 5% carbon dioxide (CO_2_).

For MTT viability assays, H9c2 cells were seeded at 5 × 10^3^ cells per well in 96-well plates and allowed to attach overnight (approximately 18–24 h) prior to treatment. Seeding density was optimized to ensure logarithmic growth during the treatment period and to prevent over-confluence, which may influence drug sensitivity measurements.

During long-term culture, cell morphology, growth characteristics, and viability were routinely monitored to ensure consistency across experimental groups. While spontaneous genetic and epigenetic drift cannot be completely excluded, the use of passage-matched control cells and standardized culture conditions minimizes potential confounding effects associated with extended passaging.

### 5.3. Preparation and Treatment of Drugs

Doxorubicin hydrochloride (Sigma-Aldrich, St. Louis, MO, USA; Cat. No. D1515) was dissolved in sterile water to prepare a 10 mM stock solution. A concentrated stock solution was selected to allow accurate serial dilutions while minimizing solvent volume in the final culture medium, thereby preventing unintended vehicle-related effects.

Metformin hydrochloride (Sigma-Aldrich; Cat. No. PHR1084) was prepared as a 100 mM stock solution in sterile water to enable accurate dilution across the working concentration range (0.7–2.8 mM) while maintaining minimal solvent volume. The solution was sterile filtered using a 0.22 µm syringe filter to remove particulate contaminants and ensure sterility during prolonged cell culture exposure experiments. Stock solutions were stored at 4 °C and freshly diluted in culture medium before use.

Since both doxorubicin and metformin stock solutions were prepared in sterile water, control wells received an equivalent volume of sterile water (vehicle control) to ensure that observed effects were attributable to the active compounds rather than the solvent. The final vehicle volume was kept constant across all experimental conditions.

For long-term exposure experiments, H9c2 cells were continuously cultured in complete Dulbecco’s Modified Eagle Medium (DMEM) supplemented with metformin at the indicated concentrations for a period of four months. Culture medium containing metformin was refreshed during routine medium changes, and cells were subcultured at 70–80% confluence to prevent overgrowth and maintain viability. Passage-matched untreated control cells were maintained in parallel under identical conditions without metformin to control for passage-related adaptations.

The four-month exposure duration was selected to model chronic cellular adaptation rather than an acute pharmacological response. Prolonged metformin exposure has been reported to induce sustained AMPK activation and epigenetic modulation [15]. Therefore, this extended treatment period was designed to allow stable genome-wide DNA methylation remodeling prior to WGBS analysis.

### 5.4. Treatment Design and Dose Selection for Genome-Wide DNA Methylation Analysis

WGBS was performed in control, low-dose metformin, and high-dose metformin groups to characterize genome-wide DNA methylation changes induced by chronic metformin exposure. Treatment concentrations were selected based on previously published studies in H9c2 cardiomyoblast cells reporting biologically relevant low and high metformin doses while preserving cellular viability and DNA integrity. The selected conditions ensured sufficient DNA yield and quality for high-resolution genome-wide methylation profiling while minimizing confounding effects related to excessive cytotoxicity. This design enabled robust comparison of global DNA methylation patterns between treatment groups and supported downstream analyses, including principal component analysis (PCA), differential methylation analysis, and pathway enrichment analysis.

### 5.5. DNA Extraction and Bisulfite Conversion

Genomic DNA was isolated using the QIAamp DNA Mini Kit (Qiagen, Hilden, Germany, Cat. No. 51304) according to the manufacturer’s instructions.Bisulfite conversion was performed using the EZ DNA Methylation-Gold™ Kit (Zymo Research, Irvine, CA, USA; Cat. No. D5005) that transforms unmethylated cytosines to uracil but leaves the methylated cytosines intact. Bisulfite conversion enables single-base resolution analysis of DNA methylation by converting unmethylated cytosines to uracil while preserving methylated cytosines, thereby allowing accurate genome-wide methylation profiling using WGBS.

### 5.6. Whole-Genome Bisulfite Sequencing

Genome-wide DNA methylation profiling was performed using WGBS. Bisulfite-converted DNA samples were submitted to Macrogen Inc. (Seoul, Republic of Korea) for library preparation and next-generation sequencing. Libraries were prepared according to a standard WGBS protocol and sequenced on the Illumina NovaSeq 6000 platform to generate paired-end reads. Raw sequencing data were returned in FASTQ format for downstream bioinformatics processing.

Sequencing libraries were prepared according to standard WGBS library preparation procedures and sequenced on a high-throughput platform to generate paired-end reads. This approach enables single-base resolution mapping of DNA methylation across the genome.

The process of sequencing data processing was performed using well-known bioinformatics pipelines, such as quality control, alignment of reads to the reference genome, and methylation calling. The levels of methylation were measured and condensed into genomic tiles to enable the downstream analysis, such as principal component analysis, differential methylation analysis, and pathway enrichment analysis.

Raw sequencing reads underwent quality assessment and adapter trimming prior to alignment. Clean reads were aligned to the reference rat genome (Rattus norvegicus assembly Rnor_6.0, rn6; GCA_000001895.4/GCF_000001895.5) using a bisulfite-aware alignment pipeline (e.g., Bismark or equivalent).

DNA methylation levels at each CpG site were quantified as β-values, calculated as the ratio of methylated reads to total reads at each locus (ranging from 0 to 1). CpG sites with insufficient coverage were filtered out according to predefined quality thresholds. Methylation data were subsequently aggregated into genomic tiles or regions to facilitate downstream analyses.

Quality metrics, including sequencing depth, bisulfite conversion efficiency, CpG coverage distribution, and global methylation profiles, were assessed to ensure high-quality genome-wide methylation measurements.

Differential methylation analysis between experimental groups (control, MET low-dose, and MET high-dose) was performed at both single-CpG and regional levels. Statistical modeling was applied to compare methylation levels across groups using established differential methylation frameworks (e.g., DSS, methylKit, or bsseq in R).

Regions were considered significantly differentially methylated when they met the following criteria:

- False discovery rate (FDR) < 0.05
- Absolute Δβ ≥ 0.20

PCA and hierarchical clustering based on β-values were performed to assess global methylation pattern differences between experimental groups. Heatmaps were generated using differentially methylated regions (DMRs) and visualized using the R package pheatmap.

Genes associated with significantly differentially methylated regions (DMRs) were subjected to functional enrichment analysis. Gene Ontology (GO) terms and Kyoto Encyclopedia of Genes and Genomes (KEGG) pathway analyses were performed using ClusterProfiler (version 4.2.2) in R. In addition, pathway enrichment analysis and functional annotation clustering were conducted using DAVID (Database for Annotation, Visualization and Integrated Discovery) [14].

Pathway visualization was performed using Pathview to illustrate KEGG pathway involvement of genes linked to significant DMRs. Enrichment results were considered statistically significant at adjusted *p*-value false discovery rate (FDR) correction according to the Benjamini–Hochberg method < 0.05.

WGBS data quality was evaluated using sequencing quality metrics, including CpG coverage and DNA methylation β-value distributions, to ensure the reliability of genome-wide methylation analysis [18].

### 5.7. Cell Viability and Dose–Response Statistical Analysis

MTT assay results are presented as mean ± standard deviation (SD) from four independent biological replicates (n = 4). Statistical comparisons between groups were performed using one-way analysis of variance (ANOVA) followed by Tukey’s post hoc multiple comparison test where appropriate.

For dose–response analysis, nonlinear regression curves were fitted using a four-parameter logistic (4PL) model. Comparisons of fitted logIC_50_ values between treatment groups were conducted using the extra sum-of-squares F test in GraphPad Prism (V11.0.1).

A schematic overview of the experimental design is provided in Supplementary Figure S1. No formal outlier testing was performed, and no data points were excluded from the analysis.

## 6. Conclusions

Chronic metformin exposure induces dose-dependent genome-wide DNA methylation remodeling in H9c2 cardiomyoblast cells and is associated with reduced cellular sensitivity to doxorubicin. Genome-wide profiling revealed widespread methylation changes affecting genes involved in oxidative stress, mitochondrial function, apoptosis, and chromatin regulation, suggesting that metabolic modulation can reshape the cardiac epigenetic landscape. These findings suggest a potential epigenetic link between metabolic modulation and cellular responses to chemotherapeutic stress.

## Figures and Tables

**Table 1:** Most and least epigenetically affected genes in high-dose metformin-treated H9c2 cells.

Categories	Genes	nDMRs	Methylation Direction	Biological Functions
Most affected	*GAB2*	89	Hypomethylated	Signal transduction adaptor; regulates PI3K/AKT and MAPK pathways, cell survival and proliferation
Most affected	*TENM4*	87	Hypomethylated	Neuronal development, axon guidance, and cell–cell adhesion
Most affected	*DLG2*	65	Hypermethylated	Synaptic scaffolding protein; essential for neurotransmission and synapse organization
Most affected	*NARS2*	56	Hypomethylated	Mitochondrial tRNA synthetase; required for mitochondrial protein synthesis and energy production
Most affected	*ZFP536*	47	Hypermethylated	Transcription factor involved in neuronal differentiation and brain development
Least affected	*LRRC10B*	1	Hypomethylated	Cardiac-specific protein involved in heart development and contractility
Least affected	Other single-DMR genes	1	Hypomethylated	Represent minimal epigenetic alteration with limited regulatory impact

**Table 2:** Most and least epigenetically affected genes in low-dose metformin-treated H9c2 cells.

Categories	Genes	nDMRs	Methylation Direction	Biological Functions
Most affected	*GLIS3*	1		Transcription factor regulating insulin signaling, neurodevelopment, and cell differentiation
Most affected	*ZDHHC13*	1	Hypomethylated	Palmitoyl-transferase involved in protein lipid modification and neuronal signaling
Most affected	*TCP1*	1		Molecular chaperone involved in protein folding and cytoskeleton assembly
Most affected	*GARRE1*	1		Regulates cell proliferation and cytoskeletal organization
Most affected	*TM6SF1*	1	Hypomethylated	Membrane protein associated with lipid metabolism and ER function
Least affected	Other single-DMR genes	1	Minor change	Show background-level methylation variation with negligible functional effect

## Data Availability

The original data presented in the study are openly available in the NCBI BioProject repository, accession number PRJNA1422602.

## Supplementary Materials

The following supporting information can be downloaded at: https://www.mdpi.com/article/10.3390/epigenomes10030044/s1, Figure S1: Schematic overview of the experimental design; Figure S2: Effect of metformin on doxorubicin-induced cytotoxicity in H9c2 cells; Figure S3: Quality assessment of genome-wide DNA methylation data in H9c2 cells; Figure S4: Calcium signalling pathway associated with metformin-induced epigenetic remodeling (low dose); Figure S5: Phosphatidylinositol (PI) signalling pathway associated with metformin-induced epigenetic remodeling (low dose); Figure S6: Thermogenesis and mitochondrial energy metabolism pathways associated with metformin-induced epigenetic remodeling (low dose); Figure S7: Calcium signalling pathway associated with metformin-induced epigenetic remodeling (high dose); Figure S8: Phosphatidylinositol (PI) signalling pathway associated with metformin-induced epigenetic remodeling (high dose); Figure S9: Thermogenesis and mitochondrial energy metabolism pathways associated with metformin-induced epigenetic remodeling (high dose).

## Abbreviations

The following abbreviations are used in this manuscript:

DOX	Doxorubicin
MET	Metformin
WGBS	Whole-Genome Bisulfite Sequencing
DNA	Deoxyribonucleic Acid
DMR	Differentially Methylated Region
DNMT	DNA Methyltransferase
PCA	Principal Component Analysis
FDR	False Discovery Rate
MTT	3-(4,5-dimethylthiazol-2-yl)-2,5-diphenyltetrazolium bromide assay
IC_50_	Half-Maximal Inhibitory Concentration
GO	Gene Ontology
KEGG	Kyoto Encyclopedia of Genes and Genomes
AMPK	AMP-Activated Protein Kinase
PI	Phosphatidylinositol
CRC	Chromatin Remodeling Complex
LD	Low dose
HD	High dose
