# Supplementary material for: Dose-Dependent Genome-Wide DNA Methylation Remodeling by Metformin Modulates Doxorubicin Sensitivity in Cardiac Cells
Source: Epigenomes. 2026 Jul 3;10(3):44. doi: 10.3390/epigenomes10030044 (PMC13398216; doi:10.3390/epigenomes10030044)

Genome-Wide DNA Methylation Remodeling by Metformin in H9c2 Cells and Its Association with Altered Doxorubicin Sensitivity

Mahmoud Abu Shayeb[1], Nagham N Hendi[2], Georges Nemer[3], Hana Hammad[4], Malek Zihlif[5,*], Heba Saadeh[6], and Heba Mansour[4]

[1] Department of Pathology, Microbiology and Forensic Medicine, Faculty of Medicine, The University of Jordan, Amman 11942, Jordan
[2] Department of Clinical Pharmacy & Therapeutics, Faculty of Pharmacy, Applied Science Private University (ASU), Amman 11937, Jordan
[3] College of Health and Life Sciences, Hamad Bin Khalifa University, Qatar Foundation, Doha P.O. Box 34110, Qatar
[4] Department of Biological Sciences, Faculty of Science, The University of Jordan, Amman 11942, Jordan
[5] Department of Pharmacology, Faculty of Medicine, The University of Jordan, Amman 11942, Jordan
[6] Department of Computer Science, King Abdullah II School of Information Technology, The University of Jordan, Amman 11942, Jordan
* Correspondence: Correspondence: m.zihlif@ju.edu.jo

Supplementary figures

Supplementary Figure S1

Schematic overview of the experimental design.

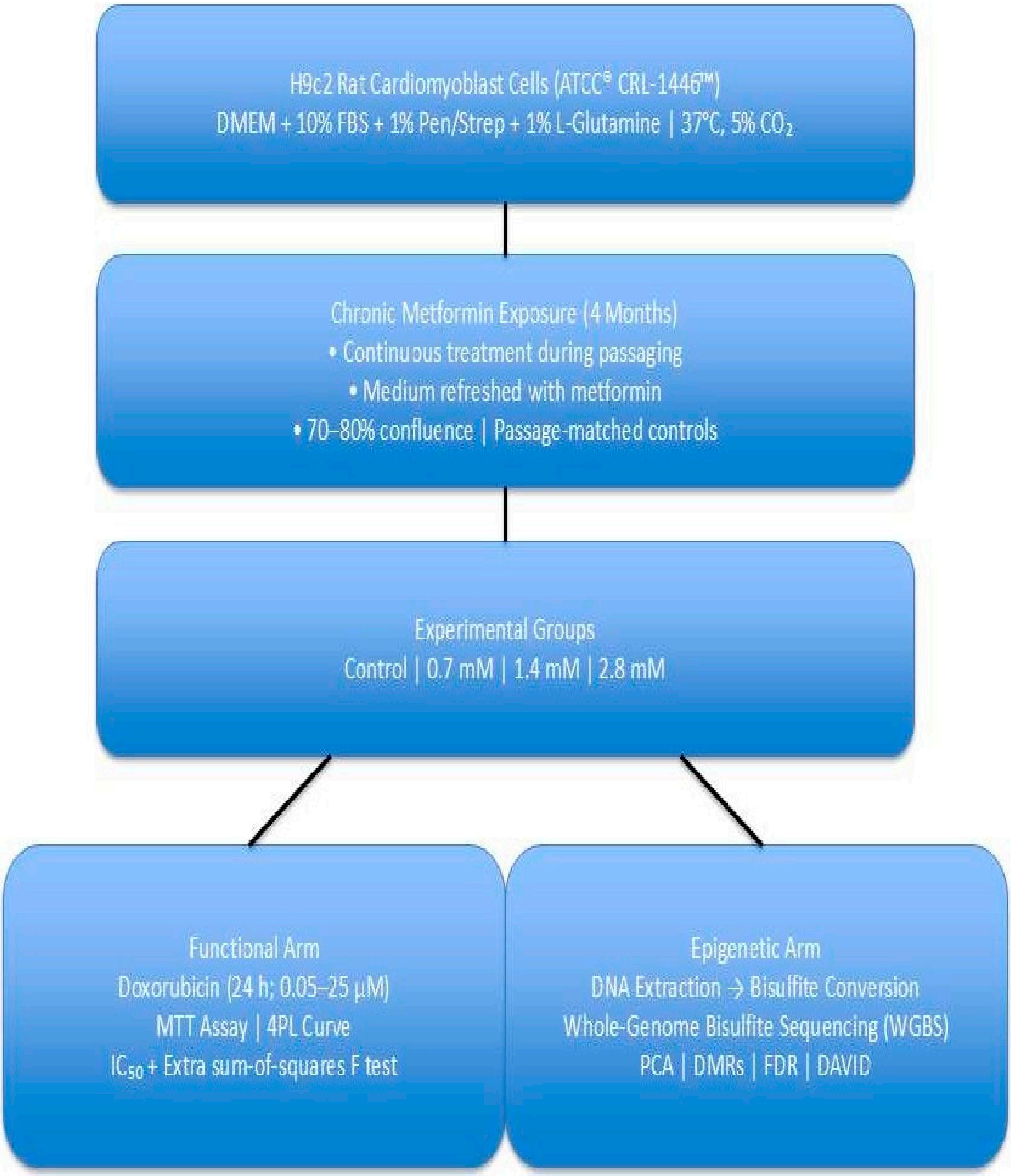

Supplementary Figure S2. Effect of metformin on doxorubicin-induced cytotoxicity in H9c2 cells.

Cell viability of H9c2 cardiomyoblast cells treated with doxorubicin (0.05 µM) in the presence of increasing concentrations of metformin (0.7, 1.4, and 2.8 mM). Viability was assessed using the MTT assay and expressed as a percentage of untreated controls. Data are presented as mean ± SD (n = 4). Statistical analysis was performed using one-way ANOVA followed by Tukey's post hoc test. No statistically significant differences (ns) were observed between DOX alone and DOX plus metformin groups at this concentration.

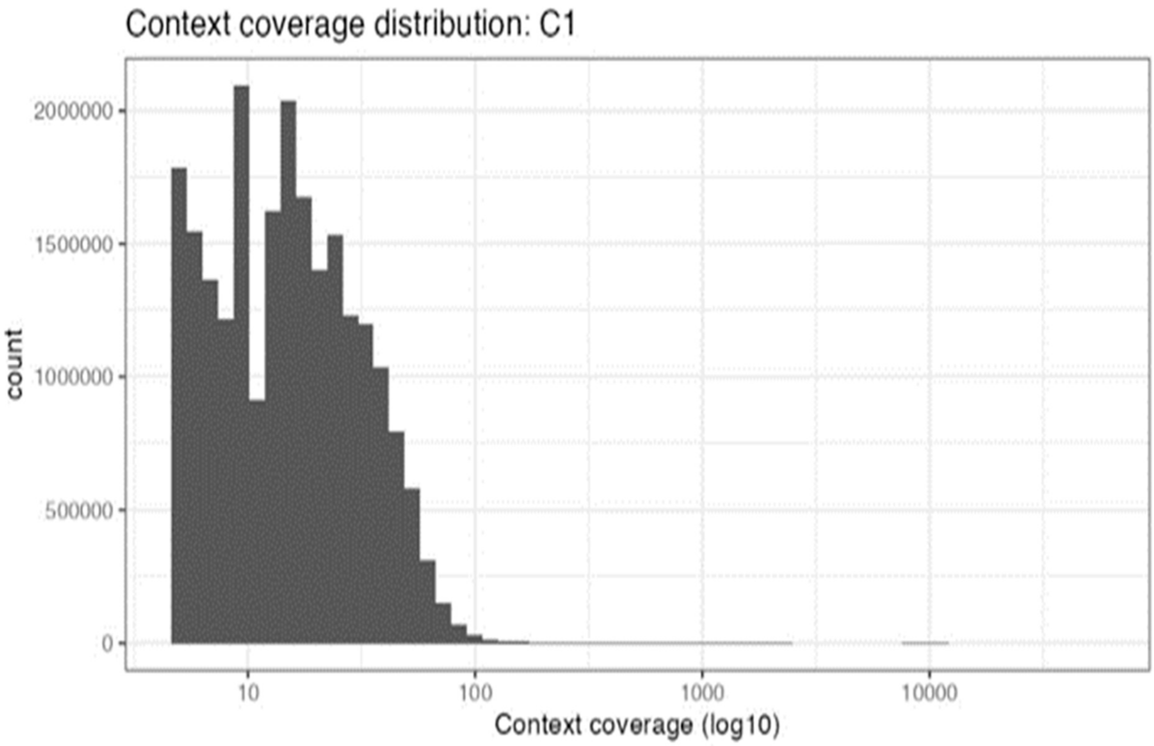

Supplementary Figure S3. Quality assessment of genome-wide DNA methylation data in H9c2 cells.

(A–C) Distribution of genome-wide DNA methylation β-values in control and metformin-treated H9c2 cardiomyoblast cells, demonstrating the expected bimodal pattern with enrichment near unmethylated (β ≈ 0) and fully methylated (β ≈ 1) CpG sites.
(D) Distribution of sequencing read coverage across CpG sites (log_{10} scale), indicating sufficient and uniform genome-wide coverage for downstream differential methylation analysis.

The preservation of the bimodal β-value distribution and adequate CpG coverage confirm efficient bisulfite conversion and high technical quality of the whole-genome bisulfite sequencing (WGBS) data.

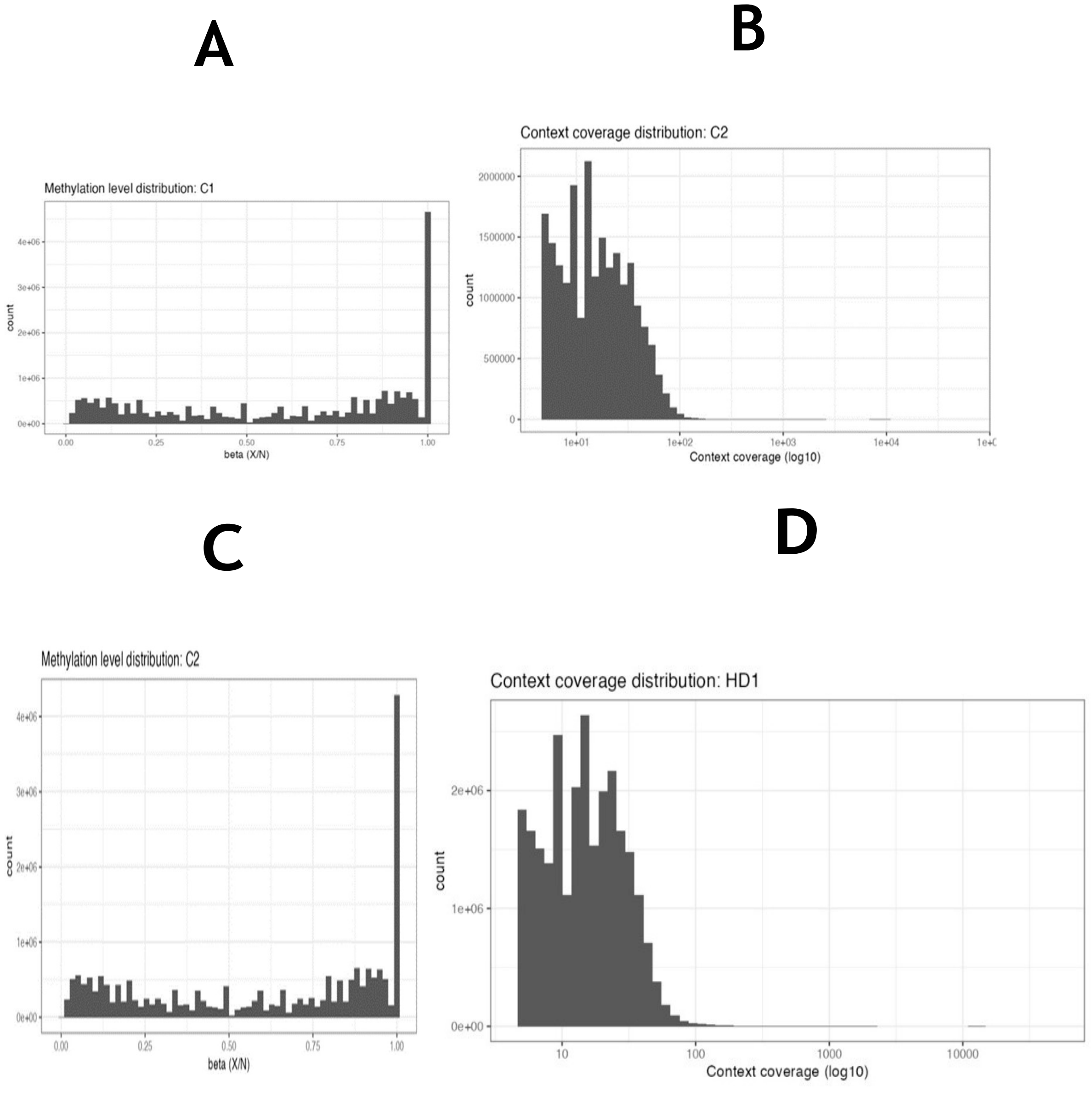

Supplementary Figure S4. Calcium signalling pathway associated with metformin-induced epigenetic remodeling (low dose).

Pathway enrichment analysis was performed using DAVID (Database for Annotation, Visualization and Integrated Discovery) [10]. The schematic representation illustrates key components of the cardiac Ca^{2+} signalling network, including Ca^{2+} influx through voltage-operated and receptor-operated channels, release from the sarcoplasmic reticulum via ryanodine receptors (RYR) and IP_3 receptors (IP3R), reuptake by SERCA, extrusion through NCX and PMCA, and mitochondrial Ca^{2+} handling via MCU and VDAC. Downstream Ca^{2+}-dependent effectors regulate contraction, metabolism, apoptosis, and transcriptional responses.

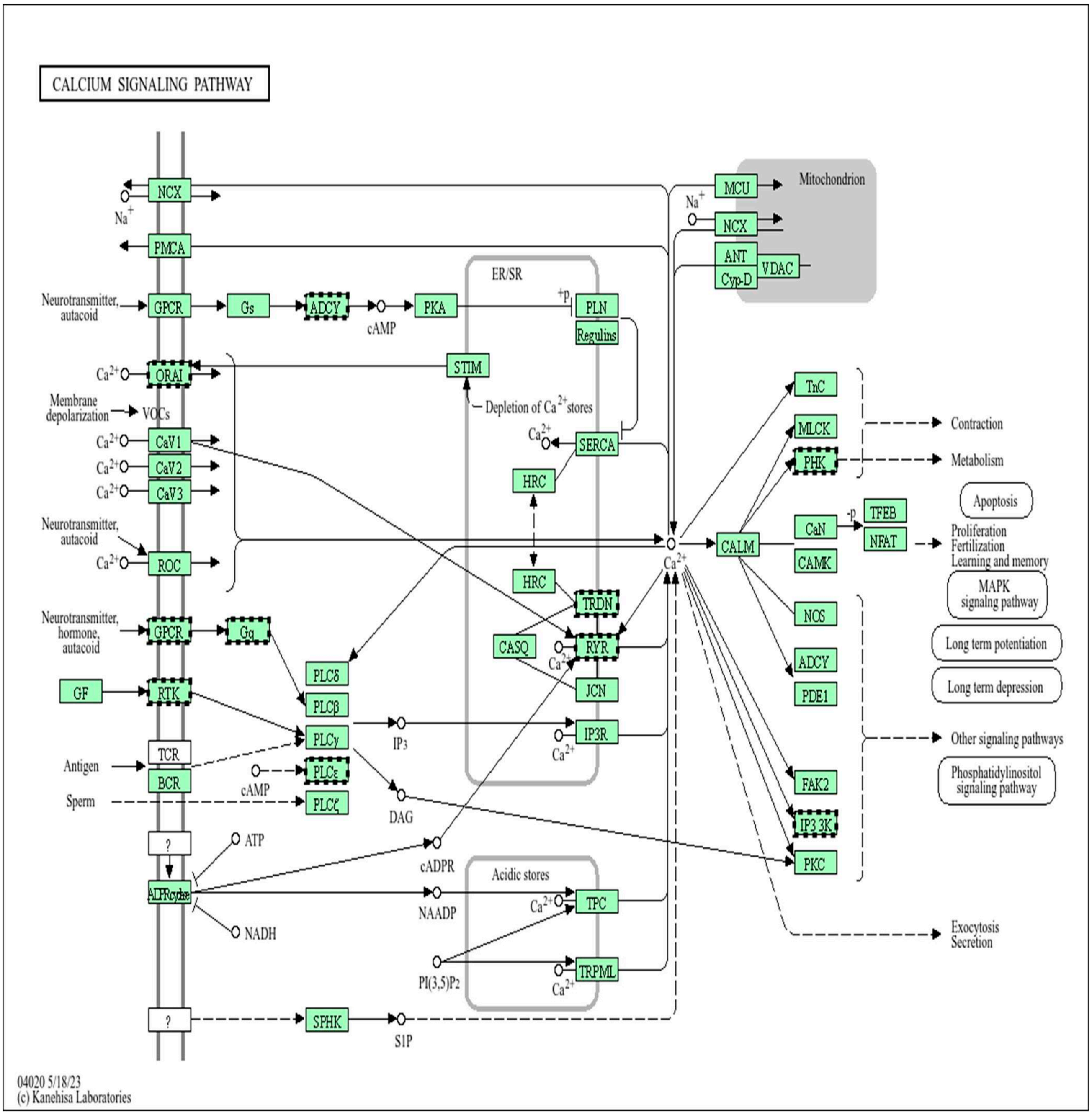

Supplementary Figure S5. Phosphatidylinositol (PI) signalling pathway associated with metformin-induced epigenetic remodeling (low dose).

Pathway enrichment analysis was performed using DAVID (Database for Annotation, Visualization and Integrated Discovery) [10]. The schematic representation illustrates the interconversion of phosphoinositides (PI, PI4P, PI(4,5)P_2, PI(3,4,5)P_3) mediated by PI kinases (PI3K, PI4K, PIP5K), phosphatases (PTEN, SHIP, INPP4), and phospholipase C (PLC). This signalling cascade generates second messengers, including IP_3 and diacylglycerol (DAG), which regulate downstream effectors such as PKC, Ca^{2+} signalling, and Akt, thereby influencing cell survival, metabolism, and stress-response pathways.

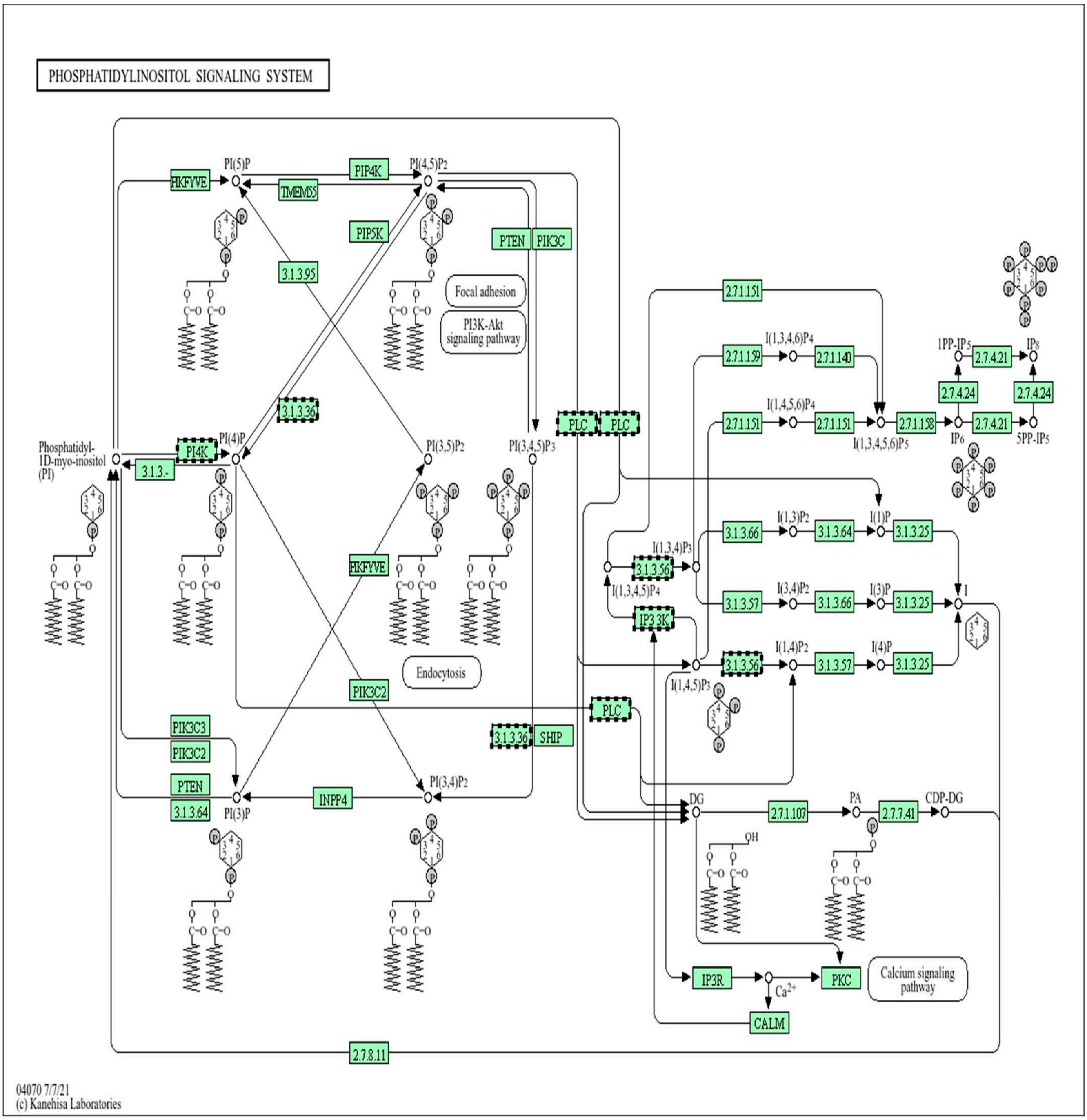

Supplementary Figure S6. Thermogenesis and mitochondrial energy metabolism pathways associated with metformin-induced epigenetic remodeling (low dose).

Pathway enrichment analysis was performed using DAVID (Database for Annotation, Visualization and Integrated Discovery) [10]. The schematic representation illustrates key regulators of mitochondrial biogenesis and energy metabolism, including β-adrenergic receptor signalling, cAMP–PKA activation, AMPK modulation, and downstream transcriptional regulators such as PGC-1α, PPARγ, and SIRT pathways. These signalling cascades coordinate fatty acid oxidation, oxidative phosphorylation, and cellular energy homeostasis.

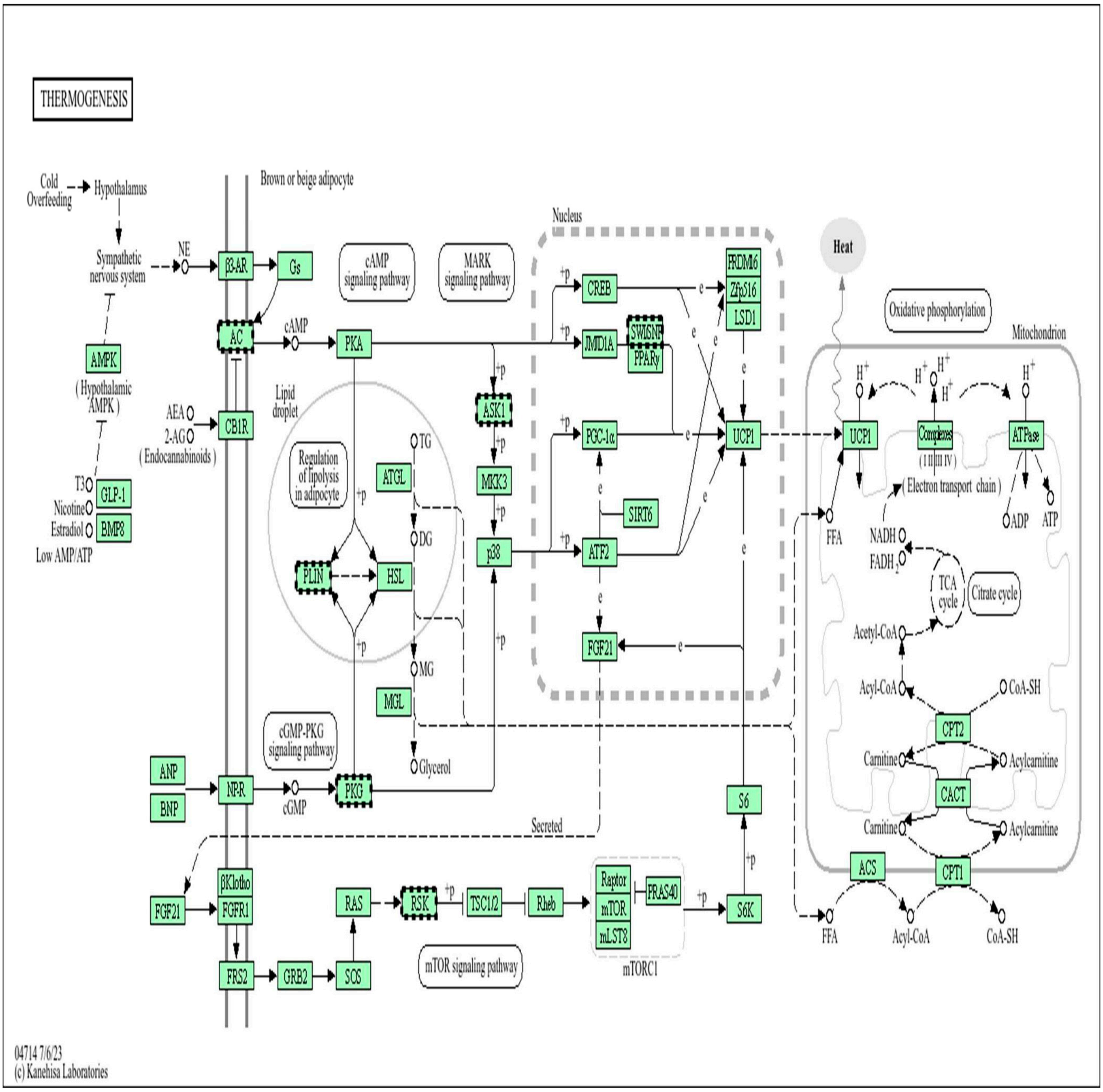

Supplementary Figure S7. Calcium signalling pathway associated with metformin-induced epigenetic remodeling (high dose).

Pathway enrichment analysis was performed using DAVID (Database for Annotation, Visualization and Integrated Discovery) [10]. The schematic representation illustrates key components of intracellular Ca^{2+} signalling, including Ca^{2+} influx channels, sarcoplasmic reticulum release mechanisms (RYR and IP_3 receptors), SERCA-mediated reuptake, mitochondrial Ca^{2+} handling, and downstream Ca^{2+}-dependent effectors regulating cellular stress responses, metabolism, and apoptosis under high-dose metformin exposure.

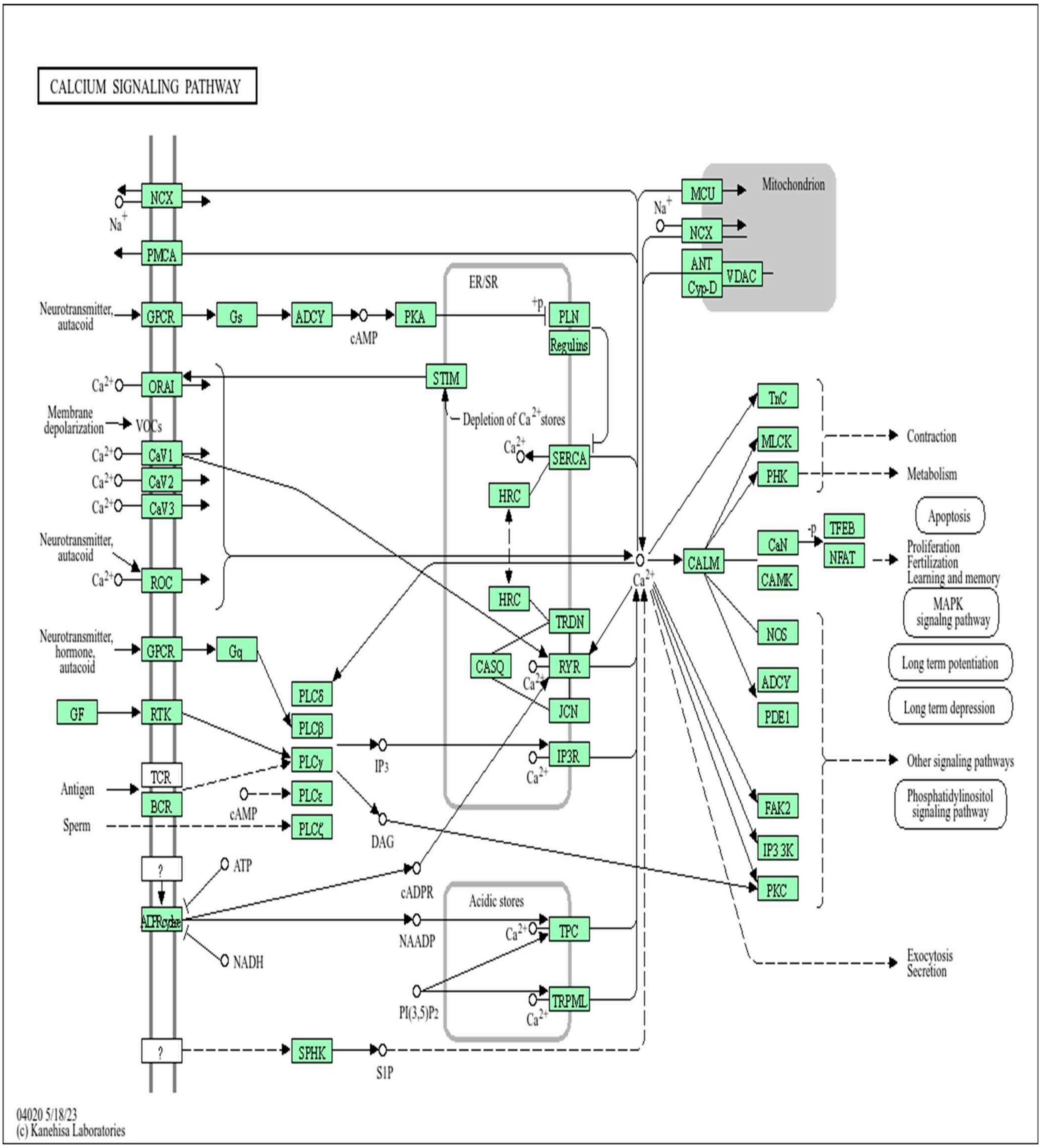

Supplementary Figure S8. Phosphatidylinositol (PI) signalling pathway associated with metformin-induced epigenetic remodeling (high dose).

Pathway enrichment analysis was performed using DAVID (Database for Annotation, Visualization and Integrated Discovery) [10]. The schematic representation illustrates the interconversion of phosphoinositides (PI, PI4P, PI(4,5)P_2, PI(3,4,5)P_3) mediated by PI kinases (PI3K, PI4K, PIP5K), phosphatases (PTEN, SHIP, INPP4), and phospholipase C (PLC). This signalling cascade generates second messengers, including IP_3 and diacylglycerol (DAG), which regulate downstream pathways such as PKC activation, Ca^{2+} signalling, and Akt-mediated survival and metabolic responses under high-dose metformin exposure.

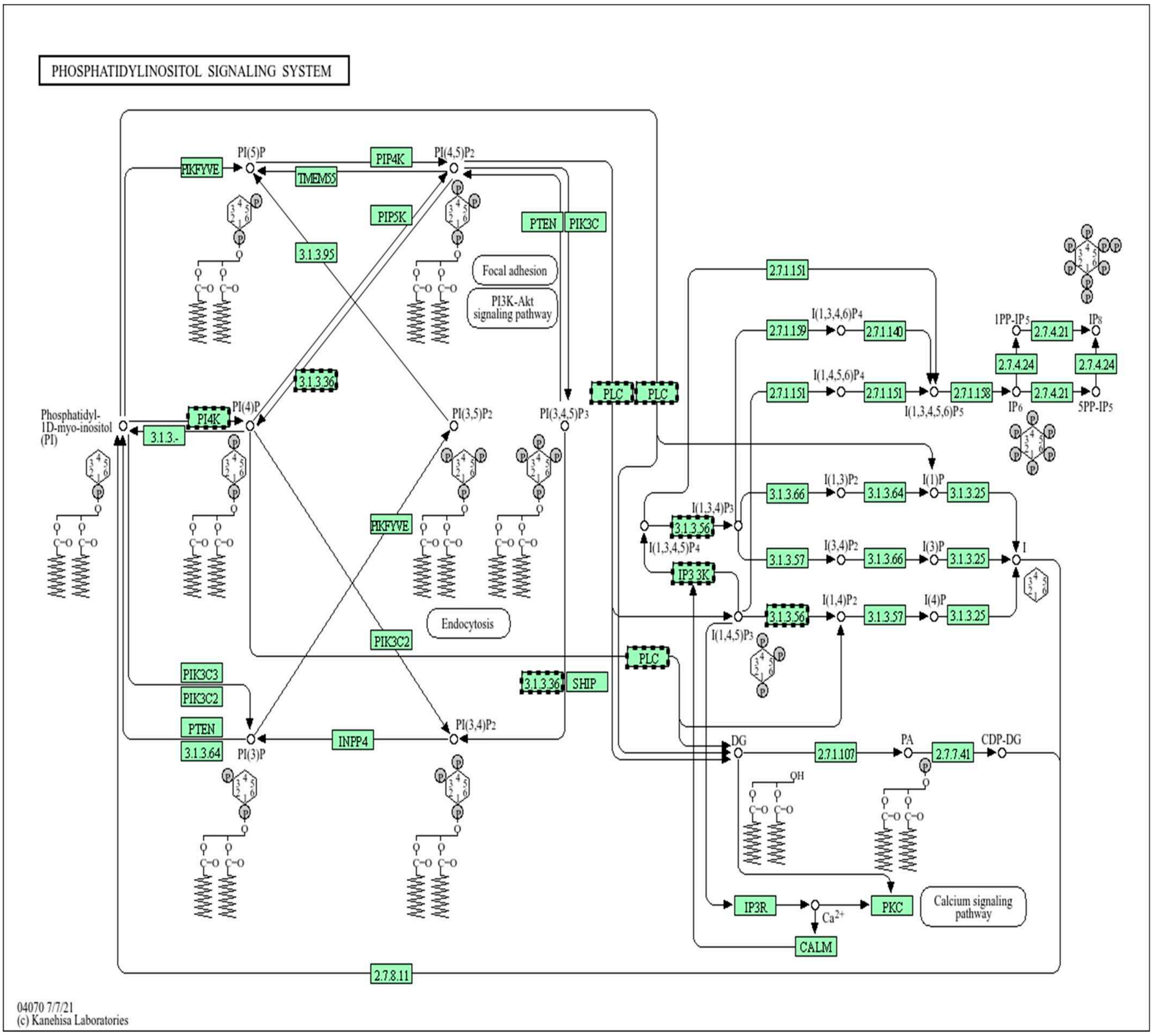

Supplementary Figure S9. Thermogenesis and mitochondrial energy metabolism pathways associated with metformin-induced epigenetic remodeling (high dose).

Pathway enrichment analysis was performed using DAVID (Database for Annotation, Visualization and Integrated Discovery) [10]. The schematic representation highlights key regulators of mitochondrial biogenesis and energy metabolism, including AMPK activation, β-adrenergic signalling, PGC-1α, SIRT pathways, and transcriptional regulators controlling oxidative phosphorylation, fatty acid oxidation, and cellular energy balance under high-dose metformin exposure.

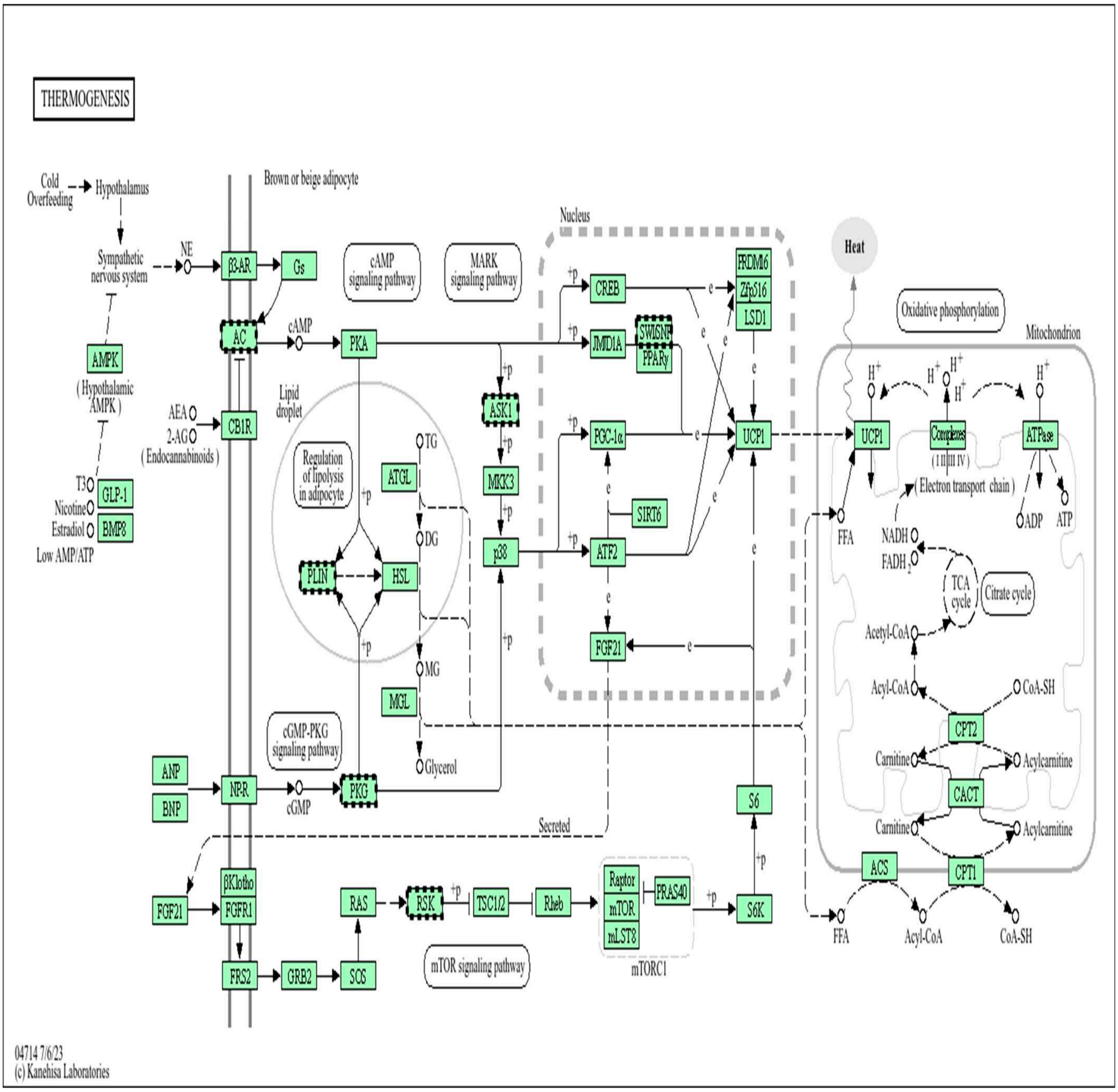

Supplement: Supplementary file 1 [file epigenomes-10-00044-s001.zip › epigenomes-4282571-supplementary.pdf]
